# Structural Characterization of the Low-Molecular-Weight Heparin Dalteparin by Combining Different Analytical Strategies

**DOI:** 10.3390/molecules22071051

**Published:** 2017-06-24

**Authors:** Antonella Bisio, Elena Urso, Marco Guerrini, Pauline de Wit, Giangiacomo Torri, Annamaria Naggi

**Affiliations:** 1 Istituto di Ricerche Chimiche e Biochimiche G. Ronzoni, 20133 Milan, Italy; urso@ronzoni.it (E.U.); guerrini@ronzoni.it (M.G.); pdewit@nl.aspenpharma.com (P.d.W.); torri@ronzoni.it (G.T.); naggi@ronzoni.it (A.N.); 2 Department of Cell and Applied Biology, Faculty of Science, Radboud University Nijmegen, 6525 HP Nijmegen, Netherlands

**Keywords:** low-molecular-weight heparin, dalteparin, NMR, LC-MS, affinity chromatography

## Abstract

A number of low molecular weight heparin (LMWH) products are available for clinical use and although all share a similar mechanism of action, they are classified as distinct drugs because of the different depolymerisation processes of the native heparin resulting in substantial pharmacokinetic and pharmacodynamics differences. While enoxaparin has been extensively investigated, little information is available regarding the LMWH dalteparin. The present study is focused on the detailed structural characterization of Fragmin^®^ by LC-MS and NMR applied both to the whole drug and to its enzymatic products. For a more in-depth approach, size homogeneous octasaccharide and decasaccharide components together with their fractions endowed with high or no affinity toward antithrombin were also isolated and their structural profiles characterized. The combination of different analytical strategies here described represents a useful tool for the assessment of batch-to-batch structural variability and for comparative evaluation of structural features of biosimilar products.

## 1. Introduction

Low molecular weight heparins (LMWHs) are heterogeneous mixtures of sulfated glycosaminoglycans with notable pharmacological activity. They were developed as alternative therapies to heparin in the prophylaxis and treatment of venous and arterial thrombotic disorders, to overcome its uncommon but potentially serious side effects, such as bleeding and thrombocytopenia, and relative unpredictability [1]. LMWHs are derived from unfractionated heparin (UFH) through controlled chemical or enzymatic depolymerization processes to yield fragments which are approximately one third the size of the original chains with Mws ranging from 1000 to 10,000 Da [2,3]. Owing to their lower molecular size they typically possess more predictable pharmacological action, better bioavailability and longer half-life [4,5]. Depending on manufacturing process, marketed LMWHs mainly differ in degree of depolymerisation, and in the chemical structure of their terminal units, as well as their therapeutic and pharmacological properties. 

Apart from characteristic terminal residues [6], their linear internal sequences are primarily composed by 1,4-linked repeating trisulfated disaccharides units containing 2-*O*-sulfated iduronic acid (I_2S_) and *N*-sulfated glucosamine 6-*O*-sulfated (A_NS,6S_). These trisulfated regions alternate with undersulfated sequences containing non-sulfated uronic acids—iduronic (I) and in lower proportion glucuronic (G)—which are usually preceded by *N*-acetylated glucosamine units (A_NAc_). The specific pentasaccharide sequence -A_NAc,6S_-G-A_NS,3S,6S_-I_2S_-A_NS,6S_-(AGA*IA), present only in some of the chains, constitutes the antithrombin binding site (AT-bs), essential for a high anticoagulant and antithrombotic activity of LMWHs, such as that of UFH. LMWH structures can also include traces of the linkage region (LR), the non-sulfated sequence G-Gal-Gal-Xyl which links the original heparin chain to the core protein of its natural proteoglycan precursor through a serine (Ser) residue [7].

Due to the structural diversity and heterogeneity arising from the different methods of preparation, the LMWHs available for clinical use are regarded as chemically and pharmacologically distinct entities, each one with its own unique efficacy and safety profile, and their therapeutic interchange is considered inappropriate. Correlating the biological properties with particular structural motifs has been the most important challenge in the design of new LMWHs as well as in the development of generic versions of these drugs [6].

For the LMWH dalteparin, approved indications include the prevention of venous thromboembolism (VTE) in patients undergoing hip replacement or abdominal surgery and in acutely ill medical patients with severely restricted mobility, in long term secondary prevention of VTE, in patients with VTE and cancer, and in acute coronary syndromes as unstable angina and non-Q wave myocardial infarction [8].

Dalteparin is produced from porcine intestinal mucosa through a relatively simple and low cost method based on a controlled deaminative cleavage with nitrous acid followed by reduction, resulting in the formation of an anhydromannitol (aM.ol) ring at the reducing end. The complete structural characterization of dalteparin, such as of all LMWHs, should supply information on the size of the chains, the monosaccharide composition in terms of iduronic/glucuronic acid, *N*-sulfated/*N*-acetylated glucosamine content and sulfation pattern and the sequence of these residues along the chains. No single technique is able to fulfil all these requirements and only a combination of orthogonal analytical approaches can provide a thorough characterization. Among the analytical approaches used for structure investigation of LMWHs, NMR spectroscopy and mass spectrometry (MS) represent the most effective techniques [3]. In particular, a growing interest has been registered in the last two decades in the application of MS, by increasingly sophisticated instruments with higher mass resolution and accuracy. A relevant contribution to the analysis of very complex mixtures such as LMWHs was provided by the direct connection of mass spectrometry to liquid chromatography systems (LC-MS) [9,10,11]. Among the most recent academic studies, fingerprint analysis by reversed-phase ion-pairing ultra-performance liquid chromatography coupled to high resolution mass spectrometry (RPIP-UHPLC–MS), has been proved to be a very important analytical method to ensure, in a very fast way, drug quality and collection of extremely interesting information about oligosaccharide structure, chain length and chemical modification [12,13]. Nevertheless, due to the extremely high complexity of samples exhibiting large polydispersity over a large molecular weight distribution together with several isomers, further approaches are necessary to obtain a detailed understanding of the samples and make ensure the comparability between different samples and lot-to-lot variability. Heparin/heparan sulfate fragment mapping by enzymatic digestion either with either heparinase I, II and III or using a mixture of them is a very common strategy reported in several studies and different analytical methods to run the depolymerized solutions and record interesting information in function to the technique used are often described [14,15,16,17,18]. Moreover, it was recently described that the quantitative NMR-HSQC analysis, applied to determine the mono- and disaccharide composition of heparin and LMWHs, can be used for detailed structural comparison studies between generic or biosimilar drugs with the reference product [19].

The present work describes the in-depth structural characterization of dalteparin by 2D-NMR and LC-MS techniques, applied both to intact chains and to their enzymatic products. The sample depolymerization by two strategies involving the exhaustive digestion with a cocktail of heparinases I, II and III and heparinase III bottom up fragment mapping, allows one to obtain samples of reduced complexity and at the same time point out eventual structural motifs. As a further level of detailed analysis, investigation of appropriate size-exclusion chromatography fractions, such as octa- and decasaccharides, and of their sub-fractions endowed of high affinity (HA) or no affinity (NA) for antithrombin was performed. The overall analytical approach here described represents an effective strategy for comparative purposes of structural features of different lots of dalteparin samples either concerning the appraisal of batch-to-batch variability or the evaluation of biosimilar products.

## 2. Results and Discussion

### 2.1. NMR Characterization

**Figure 1:**
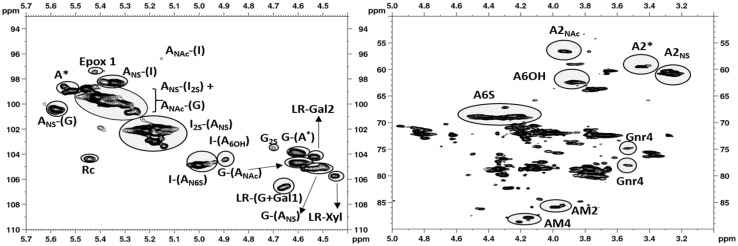
Anomeric (left) and ring (right) regions of ^1^H/^13^C HSQC NMR spectrum of Frag-5. AM corresponds to aM.ol.

The mono/disaccharide percentage content of eleven different batches of Fragmin^®^ was determined by applying a recently validated method [19]. The HSQC spectrum reported in Figure 1 shows all major and minor signals used for integration. Results are presented in Table 1.

The main structural features of dalteparin revealed by HSQC NMR analysis were the presence of an anhydromannitol ring as the only detectable residue at the reducing end, the relatively high content of sulfated monosaccharides and a substantial percentage of G-(A*) marker of the binding site for antithrombin, in agreement with previous results [20], the absence of N-acetylated glucosamine linked to non-sulfated iduronic acid [A_NAc_-(I)] and a modest presence of linkage region. Moreover, traces of anomalous structures were also detected, such as epoxide and 2-deoxy-2-C-hydroxymethylpentafuranosidic residues. The former is the result of alkaline treatments possibly suffered by the parent heparin and are identified by typical H2/C2 and H3/C3 signals at 3.74/54.2 and 3.82/53.3 ppm, respectively. Such epoxides can convert to l-galacturonic acid (GalA) during the processes for preparation of both unfractionated heparin and LMWH and this structure was identified too. The latter structure is a side effect of nitrous acid treatment of heparin used to obtain dalteparin. Actually, some internal glucosamine residues can undergo a deamination to give, by ring contraction (Rc), a 2-deoxy-2-C-hydroxymethylpentafuranosidic residue, which hexocyclic CH_2_ groups are identified by the typical chemical shift at 5.45/104.5 ppm [21].

### 2.2. Chain Mapping

**Figure 2:**
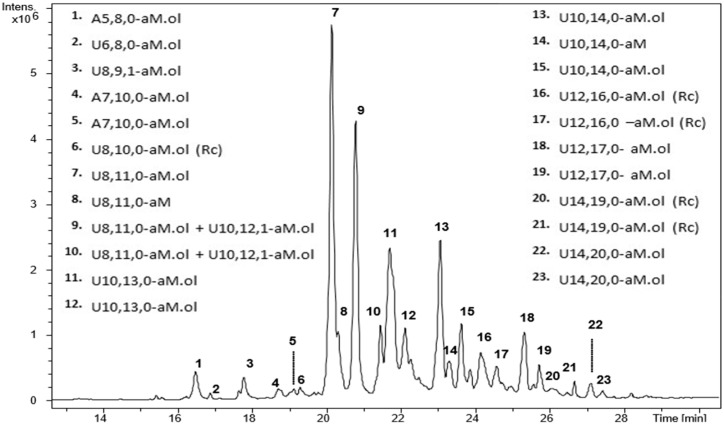
LC-MS profile (BPC) of a representative Fragmin^®^ sample (Frag-5). Mass signals assignment of the main components is reported. LC conditions: isocratic step at 15% B for 1 min, followed by a linear gradient from 15% to 40% B in 31 min; then, column washing and reconditioning in the initial conditions were performed.

The eleven Fragmin^®^ samples were subjected to LC-MS analysis of the whole intact mixture. The UHPLC method, using pentylamine as ion reagent in the ion pair reversed phase (IPRP) liquid chromatography, provided a good chromatographic separation of numerous components in a relatively short time range (less than 30 min). Mass spectral information allowed us to calculate oligosaccharide composition, in terms of chain length, number of sulfate and/or acetyl groups, and chemical modifications induced by the production process. The structure hypothesis was expressed using a code consisting of a letter indicating the non-reducing residue, such as U (uronic acid) or A (glucosamine) followed by three numbers indicating monosaccharide residues, sulfate groups and *N*-acetyl groups, respectively, followed by the aM.ol symbol for the fragment residues terminating with 2,5-anhydro-D-mannitol. All samples displayed highly similar UHPLC/MS profiles in terms of number and shape of peaks; in Figure 2 a representative base peak chromatogram (BPC) is reported. Mass spectra details of the main signals detected in all dalteparin samples are reported in Table S1.

Twentythree main species ranging from pentamer to tetradecamer were detected. It is important to underline that the intensity of peaks observable in Figure 2 is not related to the content of oligomeric species. Actually, the ion desorption efficiency in mass spectrometry ionization source is strongly affected by structure and molecular weight of components. In general, the higher the molecular weight of a species the lower the MS response is. This explains the apparent discrepancy between the LC-MS profile displayed in Figure 2 and the oligomeric composition profiles obtained by size exclusion chromatography (Section 2.5, Figures S3–S5).

The main species detected are associated to anhydromannitol derivative structures, most of them having a high degree of sulfation and no acetyl group. Almost all the oligosaccharides have a high degree of sulfation, but a few monoacetylated species were also observed. Odd species (penta- and heptasaccharides) were detected as minor components. Since they have a glucosamine residue at the non-reducing end, they represent original terminal chains of the parent heparin which dalteparin was derived from. 

Interestingly, a number of fully sulfated oligosaccharides were detected, such as U8,11,0-aM.ol, U10,14,0-aM.ol, U12,17,0-aM.ol and U14,20,0-aM.ol. They are expected to correspond to regular sequences made up of trisulfated disaccharides (I_2S_-Glc_NS,6S_) and terminating with the typical anhydromannitol moiety, i.e., (I_2S_-Glc_NS,6S_)_n_-I_2S_-aM.ol_6S_, where *n* range from 3 to 6. Nevertheless, for each of the above oligomers at least two isomers were found: for U8,11,0-aM.ol in particular, two isomers were recorded as main components under the peaks 7 and 9, and a third one as minor species in the peak 10. Different isomers could contain a G-A* sequence in different positions (positional isomers).

The molecular mass of dalteparin oligosaccharides terminating with 2,5-anhydro-D-mannitol shows a loss of 15 Da with respect to the MW of unmodified heparin oligosaccharides, corresponding to the loss of an amino group produced by the depolymerization process.

Mass signals corresponding to a further loss of 15 Da can be often observed, suggesting an additional deamination, most probably occurred on glucosamine residues within the chain, with no subsequent depolymerization. In agreement with NMR data, such signals were associated to a ring contracted (Rc) unit, derived from a glucosamine residue [21]. Structures with the Rc unit were observed in nearly all peaks of the chromatogram. As reported in Figure 2, the presence of all these structures has been verified in peaks 6, 16, 17, 20 and 21. Minor mass signals corresponding to the expected molecular mass with 2 Daltons less were detected and identified as an aldehyde form (aM) produced by an incomplete reduction to the 2,5-anhydro-D-mannitol terminal residue.

### 2.3. Building Blocks Analysis by Heparinases I, II, III Digestion

**Figure 3:**
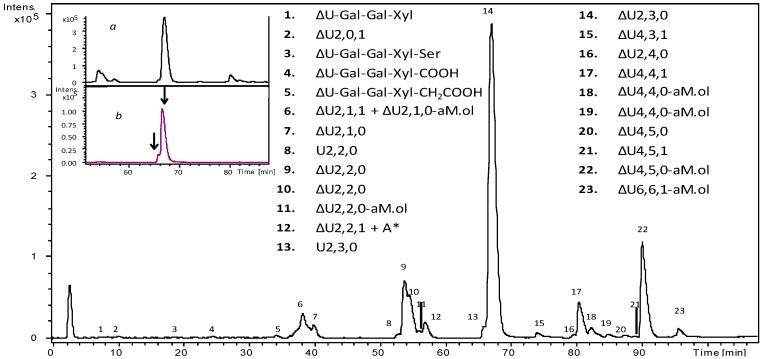
LC-MS profile (BPC) of a representative Fragmin^®^ sample (Frag-5) depolymerized by a cocktail of Heparinase I, II, III. Mass signals assignment of the main components is reported. *Inset*: expansion of chromatogram portion with the main unsaturated trisulfated disaccharide (ΔU_2S_-A_NS,6S_) eluted (panel a); extracted ion chromatograms (EIC) of *m*/*z* 594.0 attributed to saturated trisulfated disaccharide (panel b): as indicated by arrows, two positional isomers were detected. LC elution conditions: isocratic step at 2% B for 10 min, followed by a linear gradient to 60% B in 90 min and final steps of column washing and reconditioning.

Under exhaustive digestion conditions, 23 major entities were typically observed (Figure 3). Mass spectra details of the main signals detected in all dalteparin samples are reported in Table S2. Regular unsaturated disaccharides with different sulfation degree, but also monoacetylated and saturated disaccharides were observed. In particular, two isomers of the saturated disaccharide U2,3,0 were detected under the peak of the main trisulfated disaccharide ∆U2,3,0, suggesting the presence of the following isomers I_2S_-A_NS,6S_ and G-A* (Figure 3). Accordingly, a number of dalteparin chains starting with G-A* sequence at the non-reducing end are expected.

Several anhydromannitol residues were identified in different chains ranging from disaccharides to hexasaccharides. Minor signals were assigned to disaccharide ∆U2,1,0-aM.ol, ∆U2,2,0-aM.ol and to hexasaccharide ∆U6,6,1-aM.ol. The highest component in the tetrasaccharides region was attributed to ∆U4,5,0-aM.ol with five sulfate groups and the anhydro derivative at the reducing end glucosamine, followed by ∆U4,4,1 with four sulfates and an acetyl group. The former could be interpreted as ∆U_2S_-A_NS,6S_-I_2S_-aM.ol_6S_ and its survival can be explained by the presence of anhydromannitol derivative; the latter can be explained as ∆U-A_NAc,6S_-G-A*. In fact, the presence of 3-*O*-sulfate glucosamine renders the glycosidic bond between *N*-acetylated, 6-*O*-sulfated glucosamine and the unsulfated glucuronic acid impervious to the action of heparinases [22]. This effect can be used to obtain interesting structural information, mainly in the tetra-hexasaccharides region. Actually, among the minor tetrasaccharide species, two interesting structures were detected such as ∆U4,5,0 and ∆U4,5,1, both deriving from the possible fragmentation of two structural variants of the binding site. Their most probable sequence interpretations are as follows: ∆U-A_NS,6S_-G-A* and ∆U_2S_-A_NAc,6S_-G-A*, respectively.

### 2.4. Bottom up Analysis by Heparinases III Digestion

**Figure 4:**
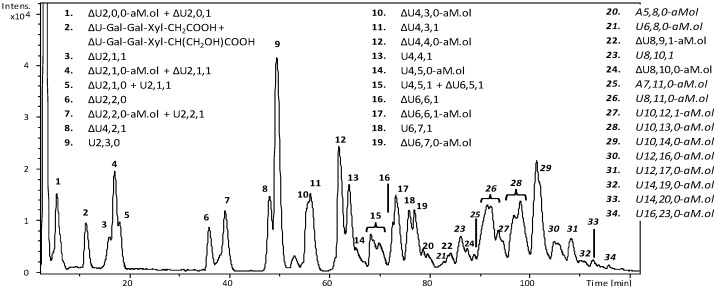
LC-MS profile (BPC) of a representative Fragmin^®^ sample (Frag-5) following digestion with heparinase III. Mass signals assignment of the main components is reported. Components identified in the parent sample, before the enzymatic digestion, are indicated in italic font. LC elution conditions: isocratic at 10% B for 5 min, linear gradient from 10% to 50% B in 55 min, linear gradient from 50% B to 90% B in 80 min, followed by column washing and reconditioning.

Heparinase III cleaves the linkage between N-acetylated glucosamine A_NAc_, with or without 6-O-sulfate, and glucuronic or iduronic acid, with preference for the former. As expected, all the fully sulfated oligosaccharides and their isomers, identified by the previous chain mapping analysis, were not recognized by this enzyme. The LC-MS profile and mass spectra assignment (Figure 4) show several oligosaccharides arising from the intact parent Fragmin^®^ (with *m*/*z* values accounting for oligomers ranging from hexa- to hexadecasaccharides). Mass spectra details of the main signals detected in all dalteparin samples are reported in Table S3.

Highly sulphated odd intact structures were also observed and identified as pentasaccharide A5,8,0-aM.ol and heptasaccharide A7,11,0-aM.ol. Confirmation of the sum formula of both oligosaccharides was provided by Ion Cyclotron Resonance-FT-MS (ICR-FT-MS) analysis (Figures S2 and S3, Supplementary Material). These unusual oligosaccharide composition can be explained by the presence of I_2S_-A* sequence inside their chain, (A_NS,6S_-I_2S_)_1or2_-A*-I_2S_-aM.ol_6OS_. Such interpretation would be in agreement with the previous building blocks results where the disaccharide ∆U2,4,0, compatible with the structure ∆U_2S_-A*, was detected.

### 2.5. Isolation of Octasaccharide and Decasaccharide Fractions

Fractionation of dalteparin by size exclusion chromatography into size homogeneous oligomeric fractions can be achieved both by Biogel P6 and Biogel P10 [20]. For the present work, all the analysed dalteparin samples underwent chromatographic fractionation on Biogel P6, to obtain a fingerprint of their overall oligomeric composition: their highly similar chromatographic profiles are compared in Figures S3–S5 (Supplementary Material). A series of peaks were resolved with each peak corresponding to a size-homogeneous oligomeric family. In particular, octa- and decasaccharide fractions were isolated from Frag-11 by implementing three chromatographic runs which yielded, after desalting, 27.6 mg of octasaccharides and 60.5 mg of decasaccharides, corresponding to 3.1% and 7.8%, respectively, of the whole sample.

### 2.6. Affinity Chromatography Separation of NA and HA Components

The isolated size-homogeneous oligosaccharide fractions were fractionated with regards to their ability to interact with AT-Sepharose column. Affinity chromatography of octa- and decasaccharides resulted in the separation of two components: the first one, considered devoid of affinity for AT as eluting at lower ionic strength (NA); the second one endowed of high affinity for AT as eluting at higher ionic strength (HA). The relative content of HA components for octa and decasaccharides with respect to the total fraction, were 5% and 6% respectively.

### 2.7. NMR Characterization of Octasaccharide and Decasaccharide Fractions and of their NA and HA Components

The isolated octa and decasaccharide fractions and their corresponding NA and HA components were studied through the quantitative compositional analysis method based on HSQC ^1^H-^13^C correlation measurement already applied for parent LMWH. The average monosaccharide content of all samples is presented in Table 2. 

**Figure 5:**
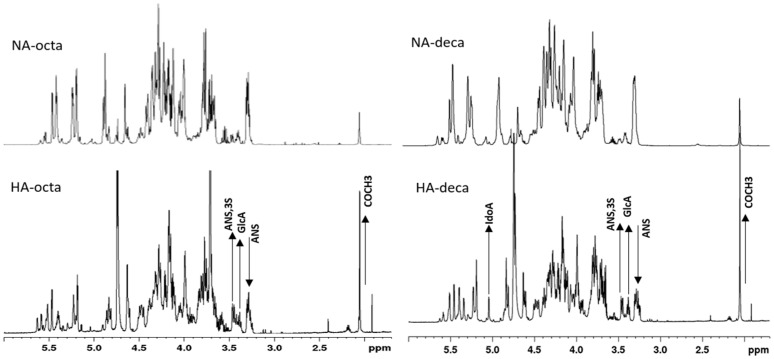
^1^H-NMR spectra of HA and NA components of octa and decasaccharide fractions. The direction of arrows, up or down, indicates the increase or decrease respectively of some characteristic signals in spectra of HA components.

Given the length of oligosaccharide sequences, additional structural information with respect to whole Fragmin^®^ samples was determined (Table 1) such as the percent content of glucuronic acid located at the non-reducing end (Gnr). Whereas no significant differences were detected between octa- and decasaccharide composition, the most important diversities were displayed by each NA and HA components with respect to the parent oligosaccharides. Signals of residues associated with the AT-binding pentasaccharide sequence, e.g., A*, G-(A*) and A_NAc_-(G), increased in HA components and their percentage content turned out to be about two-three times higher with respect to parent oligosaccharide fractions. Monodimensional ^1^H-NMR spectra of HA and NA components of the two oligosaccharide fractions clearly revealed the main structural differences above mentioned (Figure 5). In both HA sub-fractions a substantial increase of the typical signals of AT-bs (A_NS,3S_, GlcA and acetyl group) appeared accompanied by the expected decrease of N-sulfated glucosamine, A_NS_. Additionally, in HA-deca, a significant increase of non-sulfated iduronic acid, which is expected to precede the AGA*IA sequence, was also observed and quantified. No trace of linkage region was detected in parent oligosaccharides or their derived components. Interestingly, despite a small amount of ring contracted unit (Rc) and galacturonic acid were detected in total octasaccharide and decasaccharide fractions, no traces of these structures appeared in both octa-HA and deca-HA components. The presence of anomalous monosaccharide units in so short sequences would impair definitely their interaction with AT.

### 2.8. LC-MS Analysis of Octasaccharide and Decasaccharide fractions

**Figure 6:**
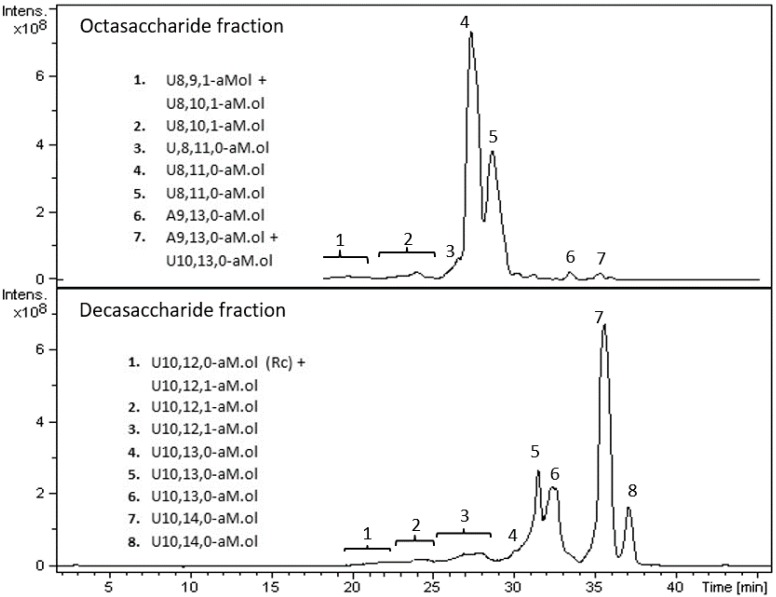
LC-MS profiles (BPC) of exemplary octasaccharide and decasaccharide fractions isolated from of Frag-5. Mass signals assignment of the main components is reported. LC elution conditions: linear gradients from 50% B to 57% B in 5 min and from 57% B to 80% B in 52 min, followed by column washing at 90% B and reconditioning in the initial conditions.

The compositional profiles of the isolated octa- and decasaccharide fractions are presented in Figure 6, together with the list of the main oligomeric species detected in BPCs. In the octasaccharide fraction, different octameric sequences were detected with a number of sulfate groups ranging from 9 to 11. In particular, nona- and decasulfated species, U8,9,1-aM.ol and U8,10,1-aM.ol respectively, contained also an acetyl group, whereas undecasulfated sequences, i.e., U8,11,0-aM.ol, accounted for a regular fully sulfated sequence. Two out of ten species are the most represented sequences, all accounting for the fully sulfated octasaccharide U8,11,0-aM.ol. A few species present in minor peaks, whose *m*/*z* ratio accounted for nona- and decasaccharides, coeluted with octasaccharides, including two isomers of a highly sulfated nonasaccharide sequence bearing 13 and 14 sulfate groups, respectively (A9,13,0-aM.ol and A9,14,0-aM.ol). As previously discussed, oligosaccharides with a glucosamine residue located at the non-reducing end represent the original terminal chains of the parent heparin.

The decasaccharide fraction turned out to be composed by eight main species bearing from 12 to 14 sulfate groups and including three monoacetylated dodecasulfated isomers. A sequence containing a ring contracted unit (Rc) was also detected. As for octasaccharide fraction, also in this case two isomers of the fully sulfated sequence U10,14,0-aM.ol were detected.

As already discussed in “Chain mapping” paragraph, together with octa and decasaccharides with regular structure (I_2S_-A_NS,6S_)_n_-I_2S_-aM.ol_6S_ (*n* = 3 or 4 respectively), isomers containing a G-A* in different positions are expected. Such oligosaccharides could either contain a fully sulfated AT-bs or a fragment if it. Actually, the presence of a G-A* located at the non-reducing terminal chain would be in agreement with the presence of Gnr detected by NMR in both oligosaccharide fractions. 

In principle, fully sulfated octa and decasaccharides could contain also G_2S_–A_NS,6S_ sequences in alternative to the prevailing trisulfated disaccharide. Nevertheless, the presence of G_2S_ residue was not detected by NMR spectra, possibly because under the sensitivity of the technique (Table 2). Further experiments are required to prove our hypothesis. Anyway, although such structural details were of great concern, their understanding did not meet the scope of the present paper.

### 2.9. LC-MS Analysis of NA and HA components of Octasaccharide and Decasaccharide fractions

It is important to underline that the sub-fractionation of oligosaccharide fractions based on their interaction with AT-Sepharose column, and then the composition of the resulting NA and HA components, greatly depend on the elution conditions applied (e.g., type of salt, salt concentration, type of gradient, etc.). 

**Figure 7:**
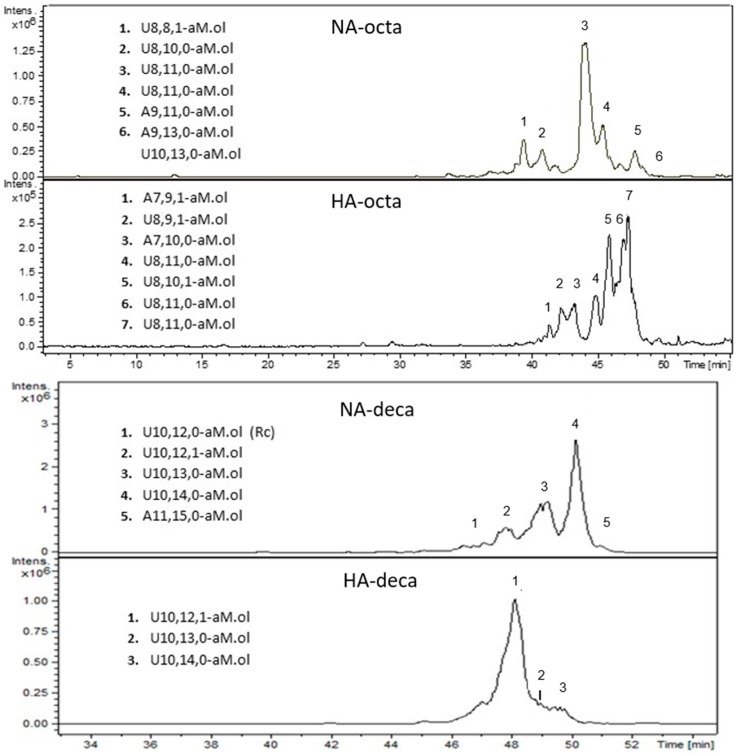
LC-MS profiles (BPC) of HA and NA components of octa- and decasaccharide fractions. Mass signals assignment of the main components is reported. LC elution conditions: isocratic at 20% Solvent B for 20 min, gradient elution from 20% B to 40% B for 10 min followed by a 20 min gradient from 40% B to 90% B; subsequently, 90% B was kept for 10 min.

The compositional profiles of the obtained NA and HA components of both octa and decasaccharides greatly differed each other for the number and intensity of peaks (Figure 7). The reduced complexity of both sub-fractions allowed to detect also some species that were not observed in the whole corresponding oligosaccharide fractions, such as A7,9,1-aM.ol and A7,10,0-aM.ol in HA-octa, U8,8,1-aM.ol in NA-octa, and A11,15,0-aM.ol in HA-deca. In HA-octa sub-fraction five main octameric species were revealed including (i) two monoacetylated sequences (U8,9,1-aM.ol; U8,10,1-aM.ol), which are expected to contain the AGA*IA sequence; (ii) three fully sulfated octasaccharides, possibly containing an AT-bs with all N-sulfated glucosamines (U8,11,0-aM.ol) [23]; (iii) two coeluted heptasaccharides, a fully sulfated one, A7,10,0-aM.ol and the monoacetylated A7,9,1-aM.ol. 

In agreement with NMR data (Table 2) and building block analysis results (Figure 3), the structure of the main species detected in HA-octa fraction can be interpreted as shown in Table 3. 

Heptasaccharides 1 and 3 are both compatible with the presence of AT-binding region, bearing an acetyl group or a sulfate group on the reducing glucosamine, respectively. Two possible interpretations have been reported for octasaccharide 2 (U8,9,1-aM.ol), both containing the AGA*IA pentasaccharide located in two different positions.

The octasaccharides U8,10,1-aM.ol, one of the most important peaks, could be explained by hypothesizing the presence of a I_2S_-A* sequence instead of G-A*, in agreement with the previous detection of ΔU2,4,0 building block. Interestingly, a synthetic octasaccharide containing the sequence I_2S_-A*-I_2S_ was found to be endowed of high binding affinity to AT [24]. As concerns the highly sulfated octasaccharide isomers U8,11,0-aM.ol two of the proposed structures contain the fully *N*-sulfated AT-binding region preceded by a 2-*O*-sulfated iduronic acid, and located in two different positions. Following cleavage with heparinase cocktail, these oligosaccharides are expected to generate the corresponding resistant tetrasaccharides U4,6,0 and ΔU4,6,0 respectively, which turned out to be almost undetectable in the whole dalteparin sample. Nevertheless, it is likely that the significant decreasing of structure polydispersity, such as in HA-Octa sub-fraction with respect to parent LMWH, allows the detection also of very minor sequences. The third undecasulfated isomer was interpreted as G-A*-I_2S_-A_NS,6S_-I_2S_-A_NS,6S_-I_2S_-aM.ol_6S_, which is in agreement with the finding of traces of non-reducing glucuronic acid in HA-Octa (Table 2). Despite such octasaccharide was expected to have a moderate AT-affinity, its presence in HA fraction is compatible with the elution conditions of AT-Sepharose column here applied, as the NA component was recovered at relatively very low salt concentration (50 mM NaCl). A possible additional or alternative interpretation of the third isomer U8,11,0-aM.ol, could be the presence of a G_2S_ instead of I_2S_, taking into account that traces of 2-*O*-sulfated glucuronic acid were detected by NMR, but further investigation is required to confirm the exact structure. In the deca-HA subfraction three main decameric species were detected: U10,12,1-aM.ol, which is supposed to contain the I-A_NAc,6S_-G-A*-I_2S_-A_NS,6S_ sequence; U10,13,0-aM.ol and U10,14,0-aM.ol, where both containing the fully N-sulfated AT-bs. As anticipated by the NMR results, the decasaccharide species containing a ring contracted unit (Rc) was recovered in the deca-NA fraction.

## 3. Materials and Methods

### 3.1. Materials

Dalteparin samples used in the present study were from different lots of injectable Fragmin^®^ (Pfizer), named as follows: Frag-1 (lot 96223A51), Frag-2 (lot 96218B51), Frag-3 (lot 96231A51), Frag-4 (lot 96238A51), Frag-5 (lot 96240B51), Frag-6 (lot 96242A51), Frag-7 (lot 96228A51), Frag-8 (lot 96235A51), Frag-9 (lot 96225A51), Frag-10 (lot 96246A51) and Frag-11 (lot Z06358). Heparinases I (EC 4.2.2.7), II and III (EC 4.2.2.8) were purchased from Grampian Enzymes (Aberdeen, Scotland, UK). Dibutylamine (>99.5%), methanol (LC-MS grade), acetonitrile (LC-MS grade), acetic acid (glacial 99.9%), formic acid (98-100%), ammonium chloride (>99.5%), potassium phosphate monobasic were purchased from Sigma-Aldrich (Milan, Italy); sodium acetate was from Merck (Milan, Italy), and calcium acetate (>97%) from BDH (VWR Milan, Italy). 

### 3.2. Fractionation by Size-Exclusion Chromatography

Fractionation by SEC to isolate octasaccharide and decasaccharide fractions was performed either on Biogel P10, as previously described [20] or Biogel P6. Briefly, on Biogel P6 column (5 × 190 cm) 300-350 mg sample dissolved in 5 mL purified water were loaded and eluted with 0.25 M NH_4_Cl at a flow rate of 1.8 mL/min. The flow-through was collected in about 8 ml fractions and their UV absorbance was detected at 210 nm. Elution profile obtained by Biogel P6 is presented in additional material. Fractions of interest were collected and pooled. The pool volume was reduced to approximately 5 mL by evaporation under reduced pressure.

### 3.3. Desalting of Oligosaccharide Fractions

Desalting was performed using TSK-gel HW40S Toyopearl (Tosoh Bioscience, Yamaguchi, Japan) column 2.6 cm × 60 cm, particle size 20–40 µm. Samples were loaded onto the column and elution was performed in 10% EtOH in water at a flow rate of 1.4 mL/min. Two-point-one ml fractions were collected using a fraction collector. Absorbance at 210 nm was evaluated for each fraction. Fractions of interest were collected, pooled and lyophilized.

### 3.4. Affinity Chromatography on AT-Sepharose

Fifteen mg of octa and decasaccharide samples were dissolved in 5 mL equilibrium buffer (Tris-HCl 50 mM, pH 7.4, NaCl 50 mM), loaded onto a 30 mL of AT-Sepharose column (2 × 9.5 cm) and eluted first with 90 ml of equilibrium buffer to recover a fraction devoid of affinity for AT (NA), then with 90 mL of Tris-HCl 0.05M pH 7.4, 2.5 M NaCl, to recover a high affinity fraction (HA), at a flow rate of 0.5 mL/min. The flow-through was collected into 26 sub-fractions, 13 for NA and 13 for HA. Each sub-fraction was analysed for the uronic acid content [25]. HA and NA fractions were desalted as previously described [20].

### 3.5. NMR

Samples (8–20 mg) were dissolved in deuterium oxide containing 0.12 mM TSP (0.6 mL). NMR spectra were measured on a Bruker AVANCE III 600 MHz spectrometer or on a Bruker AVANCE IIIHD 500 MHz spectrometer (Bruker, Karlsruhe, Germany), both equipped with a 5 mm TCI cryo-genic probe. Proton spectra were measured with water suppression (decoupling power corresponding to 5 Hz linewidth). HSQC experiments (hsqcetgpsisp2.2 Bruker pulse sequence) were performed at 303 K, by using 32 dummy scans; from 60 to 48 scans; 2 s (500 MHz) or 2.5 s (600 MHz) relaxation delay; 8 ppm (F2) and 80 ppm (F1) spectral width; transmitter offset was set at 4.7 ppm(F2) and 80 ppm (F1); 1024 points were collected in F2 and 320 increments in F1. Zero filling was applied to 4k in F2 and, in F1, linear prediction to 640 points and zero filling to 1k; a 90 shifted squared sine bell-function was applied in both dimensions. Spectra were integrated by using the standard Topspin routine using rectangular integration domains with manual adjustment of regions according data published [19].

### 3.6. Exhaustive enzymatic Depolymerization with Heparin Lyases

Heparinase I, II, III. Each sample (20 μL of a 20 mg/mL solution in water) was incubated at 25 °C for 48 h in a total volume of 160 μL, containing 20 μL hep.ase I, II and III (0.4 IU/mL of each heparinase in 10 mM K_2_HPO_4_ buffer, pH 7.0) and 120 μL of 100 mM sodium acetate buffer pH 7.0, containing 2 mM of calcium acetate and 0.1 mg/mL bovine serum albumin (BSA).

Heparinase III. Each sample (20 μL of a 20 mg/mL solution in water) was incubated at 25 °C for 48h in a total volume of 160 μL, containing 20 μL hep.ase III (0.4 IU/mL 10 mM K_2_HPO_4_ buffer, pH 7.0) and 120 μL of 100 mM sodium acetate buffer pH 7.0, containing 2 mM of calcium acetate and 0.1 mg/mL BSA.

Enzyme inactivation. At the end of each incubation, enzymes were inactivated by a 2-min heating at 100 °C, and sample solutions were filtered onto 0.22 μm membrane.

### 3.7. LC-MS Analysis

Chain mapping analyses of intact samples were run on UHPLC system (Platin Blue, Knauer, Berlin, Germany) coupled to ESI-Ion Trap mass spectrometer (amaZon SL, Bruker Daltonics, Bremen, Germany). 5 µL of sample solution, prepared at the concentration of about 5 mg/mL, was injected on C_18_ Blue Orchid (150 mm × 2.0 mm i.d., 1.8 µm particle size, Knauer) column (maintained at 40 °C) and run at the flow rate of 0.3 mL/min, by the mobile phases A (10 mM pentylamine, 10 mM acetic acid in water/acetonitrile 95:5 *v*/*v*) and B (10 mM pentylamine, 10 mM acetic acid in acetonitrile) according to the following gradient: isocratic step at 15%B for 1 min, followed by a linear gradient from 15% to 40% B in 31 min; then, column washing by a gradient until 100% B kept for 5 min and reconditioning in the initial conditions were performed. The electrospray interface was set in negative ionization mode (Spray Voltage +4200 V), to record total ion current profiles in the *m*/*z* 300–2000 mass range. Nitrogen was used as a drying (9 L/min) and nebulizing gas (30 p.s.i.) and the ion transfer capillary was kept at 200 °C.

Building blocks composition and fragment mapping analyses were run on HPLC system (Ultimate 3000, Dionex, Sunnyvale, CA, USA) connected to ESI-Q-TOF mass spectrometer (micrOTOF_Q_, Bruker Daltonics). Sample solutions at the concentration of 2 mg/mL in water were injected on C_18_ KINETEX column (100 mm × 2.1 mm i.d., with 2.6 µm particles, Phenomenex, Aschaffenburg, Germany) hold at room temperature and run by mobile phases A (10 mM dibutylamine and 10 mM acetic acid in water) and B (10 mM dibutylamine and 10 mM acetic acid in methanol) at 0.1 mL/min. Analyses of heparinases I, II, III digestion products were performed by injecting 5 µL of sample solution and eluting according to the following gradient: isocratic step at 2% B for 10 min, followed by a linear gradient to 60% B in 90 min and final steps of column washing and reconditioning. The separation method of heparinase III digestion products (injection volume of 10 µL) comprised an isocratic step of 5 min at 10% B, a linear gradient from 10% to 50% B in 55 min and a second linear gradient from 50% B to 90% B in 80 min, followed by column washing and reconditioning.

Mass spectrometry detector was set in negative polarity (Capillary voltage: +3200 V) in the mass range from *m*/*z* 200 to *m*/*z* 2000; nitrogen gas, used as nebulizer and heater gases, was set at 0.9 bar and 7.0 L/min, respectively; the ion transfer capillary was held at 180 °C.

Oligosaccharide fractions were run on Agilent 1100 HPLC system (Agilent Technologies, Santa Clara, CA, USA) coupled to ESI FT-ICR mass spectrometer (Solarix, Bruker Daltonics). Sample solutions at concentration of 10 mg/mL were injected (2 µL) on C_18_ KINETEX column (100 mm × 2.1 mm i.d., 2.6 µm partcles size, Phenomenex) held at room temperature and run by mobile phases containing 10 mM dibutylamine and 10 mM acetic acid (A: in water 100%, B: in methanol 100%) at 0.1 mL/min according to the following steps of linear gradient: from 50% B to 57% B in 5 min, from 57% B to 80% B in 52 min , from 80% B to 90% B in 16 min; then, 90% B was kept for 10 min and followed by column conditioning in the initial conditions. MS conditions were: capillary voltage at + 3200 V, nebulizer gas 1.0 bar, drying gas 3.7 liters/min, ion transfer capillary temperature 180 °C, mass range 200–3000 *m*/*z*.

Calibration of ESI-Q-TOF mass spectrometer was performed by using water -isopropanol 1:1 *v*/*v* solution containing HCOOH 0.2% and 5 mM NaOH; the ESI FT-ICR MS system was calibrated using sodium trifluoroacetate solution (0.05 mg/mL in water: acetonitrile 50:50 *v*/*v*), while low concentration tuning mix (Agilent Technologies) was employed for the mass range calibration of the ion trap detector. The LC-MS profiles and mass spectra were elaborated using the DataAnalysis software (Bruker Daltonics).

Octa-HA, octa-NA, deca-HA and deca-NA were run on an Agilent 1100 HPLC system (Agilent Technologies) coupled to an Esquire 3000 Plus (Bruker) as mass detector. Reversed-phase separation of LMWH fractions was carried out on a C_18_ KINETEX column (100 mm × 2.1 mm i.d., 2.6 µm partcles size, Phenomenex). Typically, 10 μg of each LMWH fraction was injected onto the column. The column was first eluted in isocratic conditions with 80% Solvent A (20 mM dibutylamine, 20 mM acetic acid in water) and 20% Solvent B (20 mM dibutylamine, 20 mM acetic acid in methanol) for 20 min. Then gradient elution gradient from 20% B to 40% B was applied followed for 10 min. The column was eluted at 40% B for 10 min. Subsequently, a 20 min gradient from 40% B to 90% B was applied and once 90% B was reached, this was kept for 10 min. Column conditioning for subsequent injections was established by a 5 min gradient from 90 % B to 20 % B and a 30 min equilibration at this solvent mixture. The flow rate and column temperature were maintained at 0.15 mL/min and 25 °C, respectively, throughout the run. The Chemstation software (Agilent Technologies) was used for instrument control. 

Mass spectrometric analysis were performed on an Esquire 3000 Plus electrospray ion trap (Bruker Daltonics). Acquisition parameters for ESI-ion trap mass spectrometer were (set) negative polarity, mass range 300–1000 *m*/*z*, capillary +3166 V, nebulizer gas 60.0 psi, dry gas 12.0 L/min, dry temperature 350 °C.

## 4. Conclusions

In the present work, a combination of different methods for detailed structural investigation of dalteparin was proposed. The strategy involves a first analysis of the whole samples through a bi-dimensional NMR study, to determine the molar percentage of all differently substituted glucosamine and uronic acids. In parallel, high resolution LC-MS study was performed both on the whole samples and on their fragments obtained by digestion with a cocktail of heparinases I, II, III and heparinase III alone. For a more in-depth investigation, the same combined NMR and LC-MS approach was applied also to two size homogeneous oligosaccharide fractions, precisely octa and decasaccharides, and further on their sub-fractions endowed with and devoid of affinity toward AT. High resolution LC-MS approach applied provided information that was complementary to that produced by HSQC NMR, allowing to obtain an accurate and detailed picture of the oligomeric composition of dalteparin. The application of orthogonal analytical methods to the study of size homogeneous oligomeric families and of their HA and NA components permitted to identify a number of sequences, especially among the octasaccharide components endowed of affinity to AT.

The overall analytical approach here reported represents an effective strategy for comparative studies of dalteparin samples, either concerning the assessment of batch-to-batch variability or the appraisal of biosimilar drugs.

## Figures and Tables

**Table 1:** Percent content of variously substituted glucosamine and uronic acid residues in different disaccharide sequences of Fragmin^®^ samples, together with typical linkage region residues.

	Frag-1	Frag-2	Frag-3	Frag-4	Frag-5	Frag-6	Frag-7	Frag-8	Frag-9	Frag-10	Frag-11
**Amines**											
A_NS_-(I_2S_)	55.7	56.6	55.2	55.8	57.2	58.1	56.3	57.2	59.0	55.2	55.7
A_NS_-I	7.6	5.9	7.0	7.6	6.6	6.8	6.9	7.1	6.5	7.5	8.1
A_NS_-(G)	5.9	6.0	5.8	5.9	5.9	5.6	6.0	5.9	5.7	5.6	5.6
A*	5.9	5.5	6.1	5.3	5.6	4.9	5.5	5.2	4.7	5.5	5.1
A_NAc_-(G)	9.4	9.5	9.0	9.5	9.3	9.2	9.2	9.3	8.9	9.7	9.7
A_NAc_-(I)	nd	nd	nd	nd	nd	nd	nd	nd	nd	nd	nd
A_NH2_	nd	nd	nd	nd	nd	nd	nd	nd	nd	nd	nd
A-epox	nd	nd	0.8 *	0.6 *	0.6 *	0.7 *	0.8 *	0.6 *	nd	1.1 *	nd
A-(GalA)	1.6	1.9	1.4 *	1.0 *	1.1 *	1.1 *	0.5 *	1.0 *	0.9 *	2.0	1.4
A_6S_	90.7	90.5	90.6	90.4	89.9	90.3	90.6	89.8	90.2	91.0	90.8
aM.ol	12.9	13.3	13.5	13.3	12.9	12.6	13.9	12.7	13.2	12.7	13.3
Rc	1.0 *	1.2 *	1.0 *	0.9 *	0.8 *	1.0 *	1.0 *	0.9 *	1.1 *	0.7 *	1.2 *
**Uronic acids**											
I_2S_	75.3	77.3	76.4	76.9	76.2	76.5	75.8	76.6	76.7	73.2	74.1
I-(A_6S_)	8.3	7.2	7.4	7.2	7.0	7.4	7.1	7.0	7.0	8.3	8.6
I-(A_6OH_)	0.6	0.7 *	0.8 *	1.0 *	0.7 *	0.7 *	0.9 *	0.9 *	0.7 *	0.8 *	0.9 *
G-(A*)	4.9	4.4	4.4	4.4	4.4	4.5	4.7	4.4	4.5	5.0	4.7
G-(A_NS_)	6.0	5.2	5.8	5.8	5.9	5.4	5.8	5.3	5.7	5.7	6.8
G-(A_NAc_)	3.9	4.6	3.9	4.1	4.5	4.7	4.9	4.5	5.3	4.1	3.8
Gnr	nd	nd	nd	nd	nd	nd	nd	nd	nd	nd	nd
epox	nd	nd	0.8 *	0.6 *	0.6 *	0.7 *	0.7 *	0.6 *	nd	1.0 *	nd
GalA	1.1	0.7 *	0.5*	nd	0.5 *	nd	nd	0.7 *	nd	1.7	1.1 *
**Linkage region**											
Gal1+G	1.6 *	1.2 *	1.6	2.1	2.0	1.8	2.1	2.0	1.9	1.3*	1.4
Gal2	1.3 *	1.0 *	1.3 *	1.6	1.5	1.4 *	1.6	1.4	1.6	1.2 *	1.0 *
Xyl-Ser-ox	0.8 *	0.6 *	0.8 *	0.9 *	0.9 *	1.0 *	1.0 *	0.9 *	1.1	0.6 *	0.7 *
Xyl-Ser	nd	nd	nd	nd	nd	nd	nd	nd	nd	nd	nd

nd indicates values under the limit of detection (LOD); * indicates values under the limit of quantification (LOQ); Gnr indicates glucuronic acid located at the non-reducing end.

**Table 2:** Percent content of variously substituted glucosamine and uronic acid residues in octa and decasaccharide fractions, and in their corresponding HA and NA components. n.d. = not detected.

	Octa	HA-Octa	NA-Octa	Deca	HA-Deca	NA-Deca
Amines						
A_NS_-(I_2S_)	56.6	34.5	58.6	58.1	27.0	59.8
A_NS_-I	2.8	4.1	2.3	4.6	13.8	4.1
A_NS_-(G)	4.4	10.3	2.8	5.3	9.0	5.1
A*	10.5	21.9	8.1	6.4	20.3	5.0
A_NAc_-(G)	2.2	7.2	2.0	4.4	11.6	3.3
A_NAc_-(I)	n.d.	n.d.	n.d.	n.d.	n.d.	n.d.
A_NH2_	n.d.	n.d.	0.8	n.d.	n.d.	n.d.
A-epox	n.d.	n.d.	n.d.	n.d.	n.d.	n.d.
A-(GalA)	0.9	n.d.	0.8	0.9	n.d.	n.d.
A_6S_	98.5	99.5	98.8	97.0	99.5	97.4
aM.ol	20.8	21.6	22.6	19.0	18.2	18.7
Rc	1.0	n.d.	1.0	1.3	n.d.	1.5
Uronic acids						
I_2S_	84.7	69.7	88.2	81.6	66.0	81.8
I-(A_6S_)	2.7	3.2	2.5	4.7	14.2	3.9
I-(A_6OH_)	n.d.	n.d.	n.d.	0.7	n.d.	0.6
G-(A*)	8.2	22.6	6.1	5.8	17.5	5.0
G-(A_NS_)	2.9	4.4	2.4	4.8	2.3	5.1
G-(A_NAc_)	0.6	n.d.	n.d.	1.5	n.d.	2.1
Gnr	4.6	traces	4.2	1.5	traces	2.9
G_2S_	n.d.	traces	n.d.	n.d.	n.d.	n.d.
epox	n.d.	n.d.	n.d.	n.d.	n.d.	n.d.
GalA	0.9	n.d.	0.7	0.9	n.d.	1.4

**Table 3:** Structure assignment of species detected in octa-HA sub-fraction.

Peak	Composition	Saccharide Sequence
1	A7,9,1-aM.ol	A_NAc,6S_-G-A*-I_2S_-A_NS,6S_-I_2S_-aM.ol_6S_
2	U8,9,1-aM.ol	I-A_NAc,6S_-G-A*-I_2S_-A_NS,6S_-I_2S_-aM.ol_6S_ and/or I_2S_-A_NS,6S_-I-A_NAc,6S_-G-A*-I_2S_-aM.ol_6S_
3	A7,10,0-aM.ol	A_NS,6S_-G-A*-I_2S_-A_NS,6S_-I_2S_-aM.ol_6S_
5	U8,10,1-aM.ol	I_2S_-A_NS,6S_-I-A_NAc,6S_-I_2S_-A*-I_2S_-aM.ol_6S_
4, 6, 7	U8,11,0-aM.ol	I_2S_-A_NS,6S_-G-A*-I_2S_-A_NS,6S_-I_2S_-aM.ol_6S_ I_2S_-A_NS,6S_-I_2S_-A_NS,6S_-G-A*-I_2S_-aM.ol_6S_ G-A*-I_2S_-A_NS,6S_-I_2S_-A_NS,6S_-I_2S_-aM.ol_6S_

## Supplementary Materials

The following are available online. Figure S1: LC-MS analysis of dalteparin Frag-5 following heparinase III digestion: confirmation of A5,8,0-aM.ol composition by ICR-FT-MS. Figure S2: LC-MS analysis of dalteparin Frag-5 following heparinase III digestion: confirmation of A7,11,0-aM.ol composition by ICR-FT-MS. Figure S3: Elution profiles on Biogel P6 of dalteparin samples Frag-1–Frag-4. Figure S4: Elution profiles on Biogel P6 of dalteparin samples Frag-5–Frag-8. Figure S5: Elution profiles on Biogel P6 of dalteparin samples Frag-9–Frag-11. Table S1: Oligosaccharide family identified by LC-MS chain mapping in all the analysed dalteparin samples Frag-1–Frag-11: mass signal assignment. Table S2: Oligosaccharide fragments produced by heparinases I, II, III digestion observed in all the analysed dalteparin samples Frag-1–Frag-11: mass signal assignment. Table S3: Oligosaccharide fragments produced by heparinase III digestion observed in all the analysed dalteparin samples Frag-1–Frag-11: mass signal assignment. (*) In blue, species observed in the untreated sample too.
