# Supplementary material for: Structural Characterization of the Low-Molecular-Weight Heparin Dalteparin by Combining Different Analytical Strategies
Source: Molecules. 2017 Jun 24;22(7):1051. doi: 10.3390/molecules22071051 (PMC6152074; doi:10.3390/molecules22071051)
Supplement: Supplementary file 1 [file molecules-22-01051-s001.pdf]

SUPPLEMENTARY MATERIAL

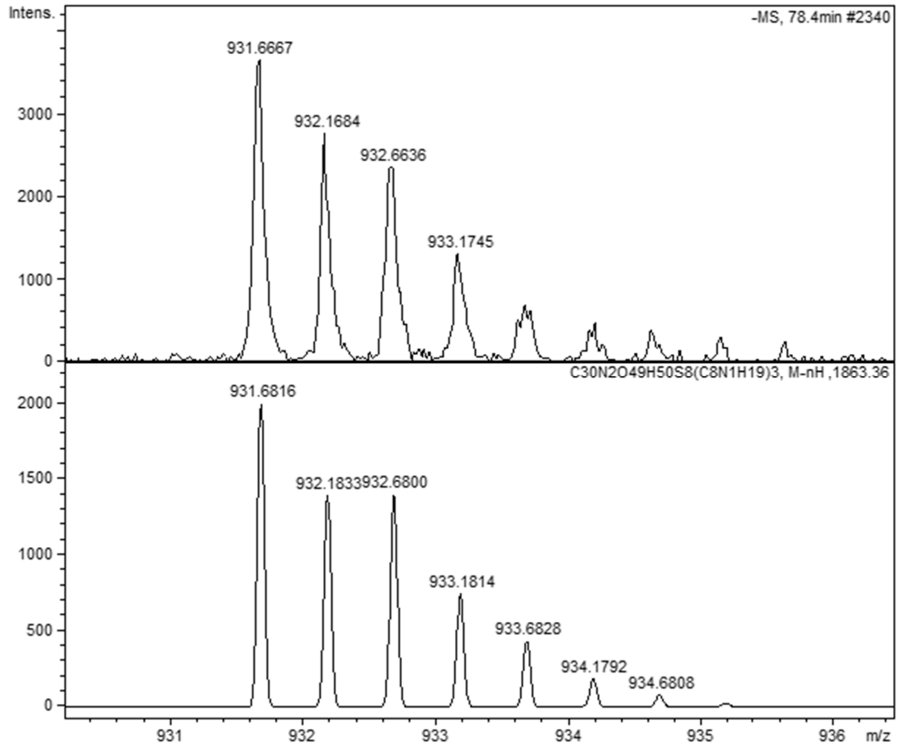

Figure S1. LC-MS analysis of dalteparin Frag-5 following heparinase III digestion: confirmation of A5,8,0-aM.ol composition by ICR-FT-MS.

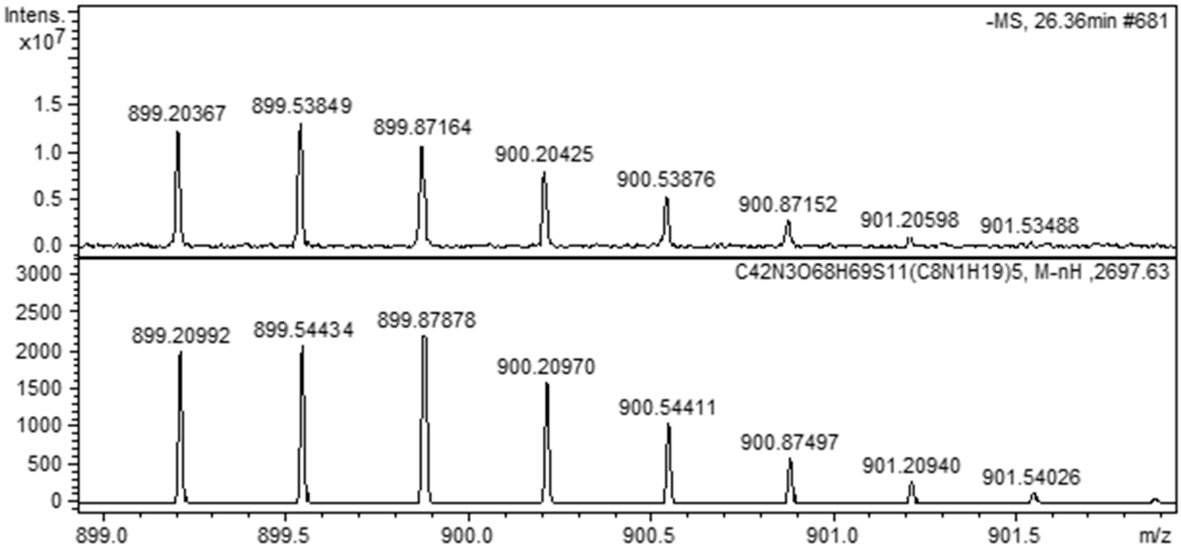

Figure S2. LC-MS analysis of dalteparin Frag-5 following heparinase III digestion: confirmation of A7,11,0-aM.ol composition by ICR-FT-MS.

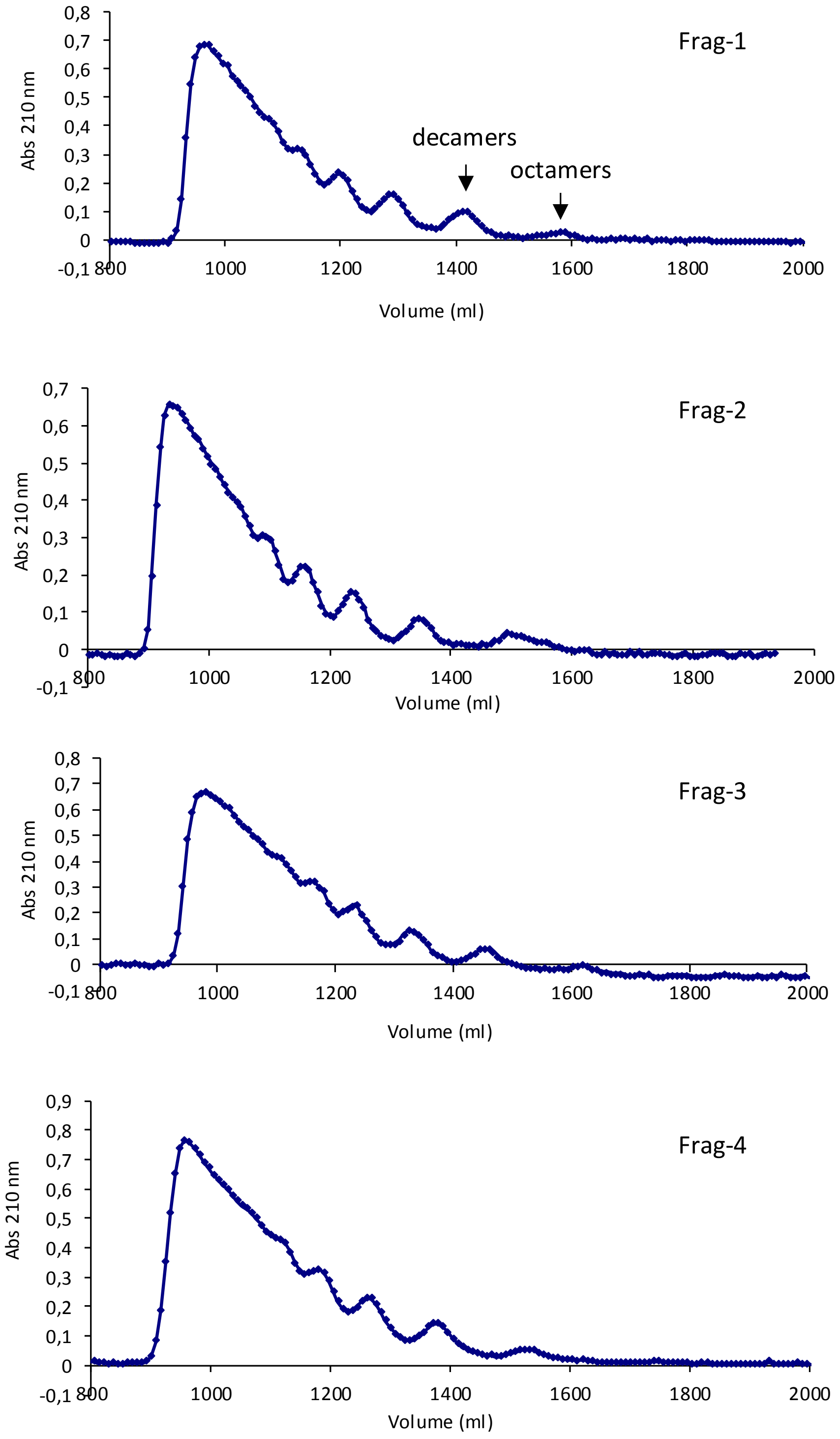

Figure S3. Elution profile on Biogel P6 of dalteparin samples Frag-1 – Frag-4.

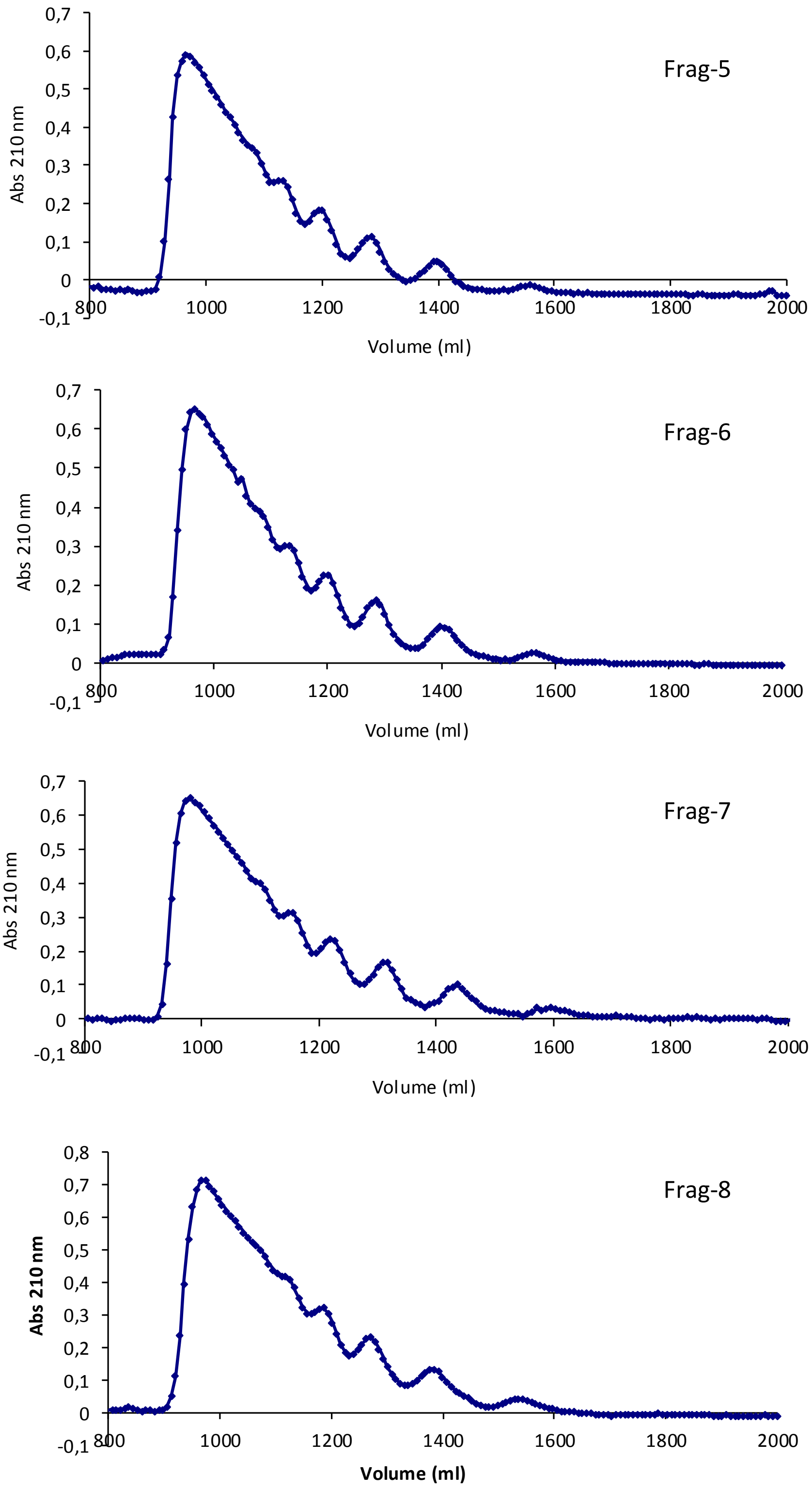

Figure S4. Elution profile on Biogel P6 of dalteparin samples Frag-5 – Frag-8.

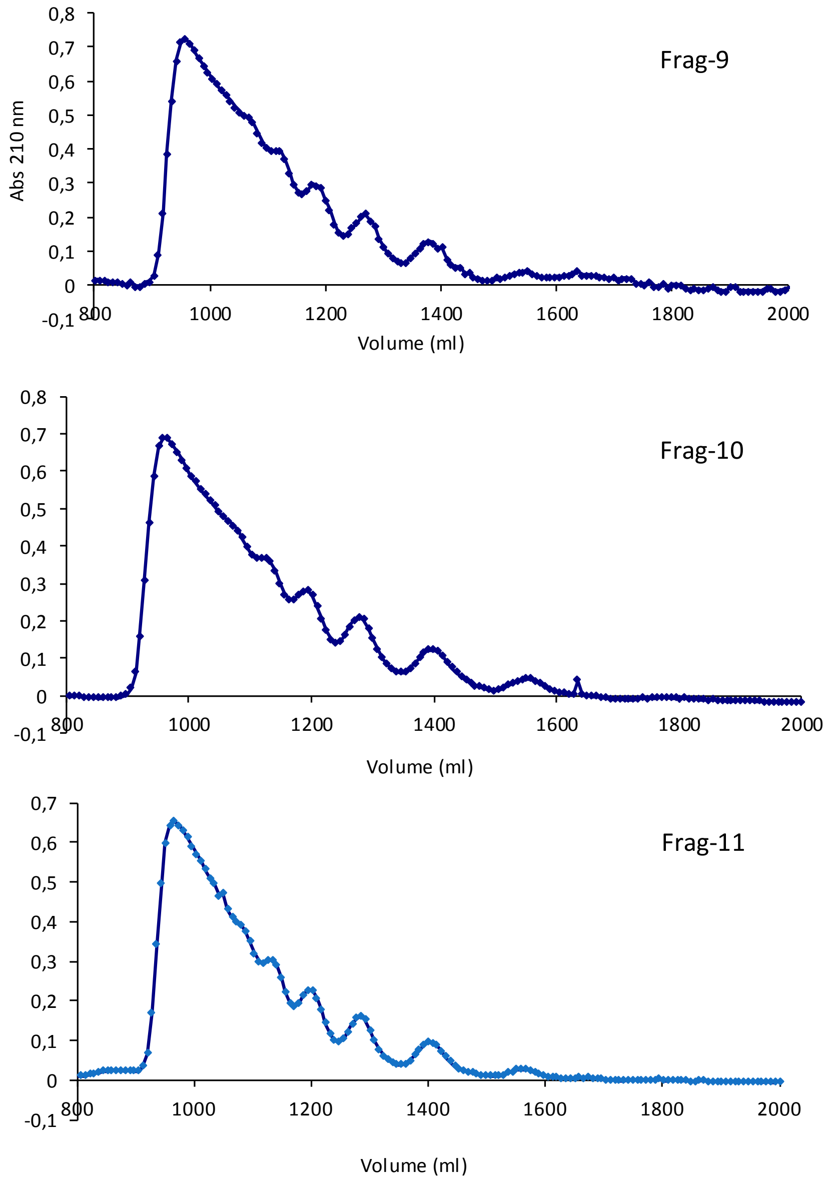

Figure S5. Elution profile on Biogel P6 of dalteparin samples Frag-9 – Frag-11.

Table S1. Oligosaccharide family identified by LC-MS chain mapping in all the analysed dalteparin samples Frag-1 – Frag-11: mass signal assignment.

Chromatographic Peak Number	Main signal (m/z)	Signal charge (z)	Pentylamine adducts	Mw	Mass signals assignment
1	912.0	-2	4	1478	A5,8,0-aM.ol
2	1000.0	-2	4	1654	U6,8,0-aM.ol
3	819.5	-3	4	2113	U8,9,1-aM.ol
4	831.5	-3	6	1975	A7,10,0-aM.ol
5	831.5	-3	6	1975	A7,10,0-aM.ol
6	832.0 + 827.0	-3	4	2151 + 2136	U8,10,0-aM.ol + Rc
7	887.5	-3	5	2231	U8,11,0-aM.ol
8	829.0	-3	3	2229	U8,11,0-aM
9	887.5	-3	5	2231	U8,11,0-aM.ol
	+ 1099.0	-3	7	2690	+ U10,12,1-aM.ol
10	887.5	-3	5	2231	U8,11,0-aM.ol
	+ 1099.0	-3	7	2690	+ U10,12,1-aM.ol
11	1082.5	-3	6	2728	U10,13,0-aM.ol
12	1082.5	-3	6	2728	U10,13,0-aM.ol
13	1167.0	-3	8	2808	U10,14,0-aM.ol
14	1166.5	-3	8	2806	U10,14,0-aM
15	1167.0	-3	8	2808	U10,14,0-aM.ol
16	1021.0 + 1017.5	-4	9	3305 + 3290	U12,16,0-aM.ol +Rc
17	1021.0 + 1017.5	-4	9	3305 + 3290	U12,16,0 –aM.ol +Rc
18	1041.0	-4	9	3385	U12,17,0- aM.ol
19	1041.0	-4	9	3385	U12,17,0- aM.ol
20	1209.0 + 1205.3	-4	11	3882 + 3867	U14,19,0-aM.ol + Rc
21	1209.0 + 1205.3	-4	11	3882 + 3867	U14,19,0-aM.ol + Rc
22	1229.0	-4	11	3962	U14,20,0-aM.ol
23	1229.0	-4	11	3962	U14,20,0-aM.ol

Nomenclature of mass signals assignment: The letter preceding the numeric code represents the kind of non-reducing end residue: U (uronic acid) and A (glucosamine), followed by the number of monosaccharide residues, sulfate groups, and N-acetyl groups, respectively. the Δ symbol represents the unsaturation on uronic acid produced by the enzymatic digestion, while the a-M.ol symbol is added for the fragment residues terminating with 2,5-anhydro-D-mannitol.

Table S2. Oligosaccharide fragments produced by heparinases I, II, III digestion observed in all the analysed dalteparin samples Frag-1 – Frag-11: mass signal assignment.

Chromtographic Peak Number	Main signal (m/z)	Signal charge	Dibutylamine adducts	Mw	Mass signal assignment	Structure identification by commercial disaccharide standards
1	631.2	-1	-	632.2	ΔU-Gal-Gal-Xyl	-
**2**	378.1	-1	-	379.1	ΔU2,0,1	ΔU-A_{NAc}
3	718.2	-1	-	719.2	ΔU-Gal-Gal-Xyl-Ser	-
4	676.2	-1	-	677.2	ΔU-Gal-Gal-Xyl-COOH	-
5	689.2	-1	-	690.2	ΔU-Gal-Gal-Xyl-CH_2COOH	-
6	458.0	-1	-	459.0	ΔU2,1,1	ΔU_{2S}-A_{Nac}
	+ 401.0	-1	-	402.0	+ ΔU2,1,0-aM.ol	-
7	416.0	-1	-	417.0	ΔU2,1,0	ΔU-A_{NS}
8	514.0	-1	-	515.0	U2,2,0	-
9	496.0	-1	-	497.0	ΔU2,2,0	ΔU-$A_{NS,6S}$
10	496.0	-1	-	497.0	ΔU2,2,0	ΔU_{2S}-A_{NS}
11	481.0	-1	-	482.0	ΔU2,2,0-aM.ol	-
12	538.0	-1	-	539.0	ΔU2,2,1	ΔU_{2S}-$A_{Nac,6S}$
	+ 418.0	-1	-	419.0	+ A*	-
13	594.0	-1	-	595.0	U2,3,0	-
14	576.0	-1	-	577.0	ΔU2,3,0	ΔU_{2S}-$A_{NS,6S}$
15	477.0	-2	-	956.0	ΔU4,3,1	-
16	785.0	-1	1	657.0	ΔU2,4,0	-
17	517.0	-2	-	1036.0	ΔU4,4,1	-
18	488.5	-2	-	979.0	ΔU4,4,0-aM.ol	-
19	488.5	-2	-	979.0	ΔU4,4,0-aM.ol	-
20	536.0	-2	-	1074.0	ΔU4,5,0	-
21	621.5	-2	1	1116.0	ΔU4,5,1	-
22	593.0	-2	1	1059.0	ΔU4,5,0-aM.ol	-
23	887.2	-2	2	1518.0	ΔU6,6,1-aM.ol	-

Table S3. Oligosaccharide fragments produced by heparinase III digestion observed in all the analysed dalteparin samples Frag-1 – Frag-11: mass signal assignment. (*) In blue, species observed in the untreated sample too.

Chromatographic Peak Number	Main signal (m/z)	Signal charge	Dibutylamine adducts	Mass signals assignment (*)
1	321.0	-1	-	ΔU2,0,0-aM.ol
	378.0	-1	-	ΔU2,0,1
2	344.0	-2	-	ΔU-Gal-Gal-Xyl-CH_2COOH
	587.0	-1	-	ΔU-Gal-Gal-Xyl-CH(CH_2OH)COOH
3	458.0	-1	-	ΔU2,1,1
4	401.0	-1	-	ΔU2,1,0-aM.ol
	458.0	-1	-	ΔU2,1,1
5	417.0	-1	-	ΔU2,1,0
	477.0	-1	-	U2,1,1
6	497.0	-1	-	ΔU2,2,0
7	482.0	-1	-	ΔU2,2,0-aM.ol
	557.0	-1	-	U2,2,1
8	437.0	-2	-	ΔU4,2,1
9	594.0	-1	-	U2,3,0
10	448.5	-2	-	ΔU4,3,0-aM.ol
11	477.0	-2	-	ΔU4,3,1
12	488.5	-2	-	ΔU4,4,0-aM.ol
13	526.0	-2	-	U4,4,1
14	537.5	-2	-	U4,5,0-aM.ol
15	566.0	-2	-	U4,5,1
	725.5	-2	-	ΔU6,5,1
16	765.5	-2	-	ΔU6,6,1
17	887.0	-2	2	ΔU6,6,1-aM.ol
18	943.5	-2	2	U6,7,1
19	906.0	-2	2	ΔU6,7,0-aM.ol
20	931.5	-2	3	A5,8,0-aMol
21	1019.5	-2	3	U6,8,0-aM.ol
22	783.3	-3	2	ΔU8,9,1-aM.ol
23	821.0	-3	2	U8,10,1
24	796.0	-3	2	ΔU8,10,0-aM.ol
25	1413.9	-2	6	A7,11,0-aM.ol
26	871.6	-3	3	U8,11,0-aM.ol
27	1110.6	-3	5	U10,12,1-aM.ol
28	1123.3	-3	5	U10,13,0-aM.ol
29	1193.0	-3	6	U10,14,0-aM.ol
30	1445.0	-3	8	U12,16,0-aM.ol
31	1514.3	-3	9	U12,17,0-aM.ol
32	1766.5	-3	11	U14,19,0-aM.ol
33	1836.2	-3	12	U14,20,0-aM.ol
34	1521.0	-4	12	U16,23,0-aM.ol
